# Mortality Trends Among US Adults With Obesity and Hypertensive Diseases Before and During the COVID-19 Pandemic (1999-2020)

**DOI:** 10.7759/cureus.110451

**Published:** 2026-06-08

**Authors:** Noor Fatima, Ibrahim Zulfiqar Ali, Talha Khalid, Suleman Khan, Eemahn Akhtar, Hafsa I Sair, Maheen Hassan, Saif Ali Malik

**Affiliations:** 1 Medicine, Shifa College of Medicine, Islamabad, PAK

**Keywords:** age-adjusted mortality rates, cdc wonder, covid-19 pandemic, demographic differences, geographic variation, health disparities, hypertension, mortality trends, obesity

## Abstract

Background

Obesity is a global epidemic. The prevalence of obesity has significantly increased in recent decades and is expected to impact a large portion of the US population. Hypertension continues to be one of the most common complications associated with obesity, and the overlap between these two conditions has been growing over time. However, mortality trends in patients with obesity and hypertension have not been investigated in the literature.

Objectives

This study aimed to investigate mortality trends, stratified by sex, race, age groups, and geographic distribution, in the US population between 1999 and 2020.

Methods

Death certificates from the Centers for Disease Control and Prevention Wide-Ranging Online Data for Epidemiologic Research (CDC WONDER) were examined and analyzed between 1999 and 2020 for patients with obesity and hypertension as the contributing causes of death. The age-adjusted mortality rates (AAMRs) and annual percent changes (APCs) per 100000 people were calculated by sex, race, age group, and geographic region.

Results

Among individuals aged ≥15, a total of 294854 deaths occurred in individuals with obesity and hypertension between 1999 and 2020. The overall AAMR increased from 1.08 in 1999 to 12.14 in 2020. The AAMR has steadily increased since 1999, with a sudden spike occurring between 2018 and 2020 during the COVID-19 pandemic. This trend has been observed across nearly all variables analyzed in our study. Our results exhibited a 28% increase in mortality related to obesity and hypertension during the early years of the COVID-19 pandemic. During the study period, men had a higher overall AAMR than women (men, 5.92; women, 4.32). Mortality was highest among the 55-74-year-old age group, followed by the 75-plus-year-old, 35-54-year-old, and finally 15-34-year-old age groups, which displayed the lowest AAMR (AAMR: 55-74, 11.76; 75+, 10.83; 35-54, 4.73; and 15-34, 0.54). Among the races, non-Hispanic (NH) Blacks had the highest overall AAMR (9.81), followed by NH American Indians or Alaskan Natives (6.01), NH Whites (4.64), Hispanics (4.08), and NH Asians or Pacific Islanders (1.17). However, NH Whites showed the highest average APC (AAPC) (11.44), indicating a possible future shift in mortality. By geographic region, the Southern United States had the highest AAMR, followed by the Western, Midwestern, and Northeastern regions. Non-metropolitan areas had consistently higher obesity- and hypertension-related AAMRs (5.63 overall) compared to metropolitan areas (4.99 overall).

Conclusion

In our retrospective analysis of death certificate data from 1999 to 2020, we found that age-adjusted mortality rates among individuals with both obesity and hypertension consistently displayed an increasing trend across all demographic groups. The overall rising AAMRs, compounded by the disproportionately high average annual percent changes among White individuals and those aged 15-34, raise serious concerns for the healthcare system. These findings have significant implications for public health policy. Focused interventions are essential to curb the upward trajectory of mortality in this population, as early intervention can greatly help tackle the dual burden of obesity and hypertension, which are largely preventable.

## Introduction

Obesity affects over one billion individuals worldwide and was associated with nearly five million deaths in 2019, making it one of the leading contributors to global mortality [1]. With a brief hiatus between 2009 and 2012, obesity prevalence in the United States has continued to rise, with projections indicating that 78% of Americans will be overweight or obese by 2030 [2]. Additionally, approximately 47.3% of adults (116 million individuals) in the United States are affected by hypertension, with rates steadily rising since 2013 [3]. Among all modifiable risk factors, hypertension contributes most significantly to the burden of cardiovascular disease in the United States [4].

The prevalence of obesity in American adults with hypertension increased from 39.6% in 2001 to 55.4% in 2023 [5]. In a study done by Forman et al., an increased BMI significantly increases the risk of developing hypertension. Obese women were found to have a 4.7 times greater incidence of hypertension compared to those with a BMI lower than 23.0 kg/m² [6].

The mechanisms underlying obesity-related hypertension are complex and involve the dysregulation of the sympathetic nervous system, insulin resistance, renal structural and hemodynamic changes, and the renin-angiotensin-aldosterone system [7]. Microcirculation changes driven by dysfunctional and inflammatory perivascular adipose tissue (PVAT) in obesity contribute to impaired vascular tone regulation and are a major factor in the development of hypertension [8].

While previous studies have examined mortality trends in patients with obesity and hypertension separately, the trends related to the combination of both conditions have not been adequately investigated in the United States. Understanding the mortality with these coexisting conditions is essential to identifying high-risk groups and guiding targeted preventative interventions. Therefore, this study aims to characterize population-level mortality trends among US adults with concurrent obesity and hypertension, stratified by sex, race/ethnicity, age group, urbanization level, and geographic region.

## Materials and methods

Study setting and population

This study utilized data from the Centers for Disease Control and Prevention Wide-Ranging Online Data for Epidemiologic Research (CDC) WONDER database, covering the period from January 1, 1999, to December 31, 2020 [9]. We analyzed death certificates from the Multiple Cause-of-Death Public Use records to identify cases involving both obesity and hypertension. Relevant deaths were defined as those in which both obesity and hypertensive diseases were listed as contributing or underlying causes of death. We identified cases of obesity using the International Statistical Classification of Diseases, Tenth Revision, Clinical Modification (ICD-10-CM) code E66 [10]. Hypertensive diseases were classified using codes I10-I15, including essential (primary) hypertension, hypertensive heart disease, hypertensive chronic kidney disease, hypertensive heart and chronic kidney disease, and secondary hypertension. The study population consisted of individuals aged 15 years and older. Mortality rates were calculated using bridged-race population estimates provided by the US Census Bureau. Data were stratified into 10-year age groups (15-24, 25-34, 35-44,…, 85+). Age-adjusted mortality rates (AAMRs) were calculated using the direct method and standardized to the 2000 US standard population. Results are reported per 100000 persons with two decimal places of precision.

Data abstraction

We gathered data on deaths associated with obesity and hypertensive diseases, which included demographic information, population sizes, the year of death, and geographic location. Data were extracted using the CDC WONDER online query system with both ICD-10 codes applied simultaneously using the AND operator, ensuring that only deaths with concurrent obesity and hypertensive disease were captured. Additional information included urban-rural classifications, states, regional categories, and the locations of death (medical facility, home, hospice, or nursing home/long-term care facility). The urban-rural classifications followed the National Center for Health Statistics Urban-Rural Classification Scheme. The geographic regions were categorized according to the US Census Bureau divisions: Northeast, Midwest, South, and West.

Race and ethnicity were recorded from death certificates, in accordance with the US Office of Management and Budget standards. Racial and ethnic groups included Hispanic, non-Hispanic (NH) White, NH Black or African American, NH American Indian or Alaskan Native, and NH Asian or Pacific Islander [11].

Statistical analysis

To assess national trends in obesity-related and hypertensive disease-related mortality, we calculated crude mortality rates and age-adjusted mortality rates (AAMRs) per 100000 population from 1999 to 2020. These rates were stratified by year, sex, race, state, and region. Crude mortality rates were determined by dividing the number of deaths related to obesity and hypertensive disease by the corresponding US population for each year. AAMRs were calculated by standardizing deaths to the 2000 US population. We reported 95% confidence intervals (CIs) for all rates. Data for NH American Indian or Alaska Native populations in 1999 were excluded from trend analyses because the death count fell below the CDC WONDER reliability threshold of 20 deaths. All other suppressed or statistically unreliable cells (flagged by CDC WONDER) were similarly excluded from subgroup trend analyses.

To analyze temporal trends, we used the Joinpoint Regression Program (National Cancer Institute), which identifies significant changes by fitting log-linear regression models across time segments [12]. To calculate the annual trends in deaths associated with obesity and hypertensive disease, we assessed the annual percent change (APC), the average annual percent change (AAPC), and relative 95% CIs. 

A trend was defined as increasing or decreasing if the slope differed significantly from zero, with significance assessed using two-tailed t-tests. A p-value of ≤0.05 was considered statistically significant.

Using AAPC numbers from 1999 to 2019, we estimated the expected AAMR for 2020, the first year of the pandemic. By comparing the estimated rate to the actual AAMR observed in 2020, we calculated the excess AAMR. To estimate the proportion of excess AAMR attributable to the pandemic, we divided the excess AAMR by the actual 2020 AAMR and reported the result as a percentage. Lastly, to estimate the extra death toll, we multiplied the actual number of deaths in 2020 by the proportion of excess AAMR.

Ethical considerations

This analysis used publicly available, de-identified data from federal databases and did not involve direct human-subject participation. As such, it was exempt from institutional review board (IRB) oversight following US federal research regulations.

## Results

A total of 294854 deaths occurred between 1999 and 2020 that were attributed to obesity and hypertensive diseases among individuals aged ≥15 years (Table 1).​​

**Table 1:** Mortality in Obese Individuals With Hypertensive Diseases, Stratified by Sex, Ethnicity, Race, Age Groups, and Census Regions in the United States (1999-2020).

Year	Overall	Male	Female	Hispanic or Latino	Not Hispanic or Latino	White	Black or African American	American Indian or Alaska Native	Asian or Pacific Islander	15-34	35-54	55-74	75+	Census Region 1: Northeast	Census Region 2: Midwest	Census Region 3: South	Census Region 4: West
1999	2365	1048	1317	115	2239	1711	629	10	15	109	817	1053	386	418	520	800	627
2000	4663	2184	2479	216	4427	3513	1092	27	31	142	1651	2062	808	813	1040	1743	1067
2001	4854	2307	2547	246	4590	3635	1167	32	20	164	1753	2127	810	838	1067	1855	1094
2002	5766	2787	2979	285	5459	4299	1389	42	36	224	2057	2550	935	950	1233	2126	1457
2003	6616	3218	3398	377	6215	4987	1534	49	46	241	2369	2977	1029	1030	1377	2552	1657
2004	7174	3584	3590	396	6754	5330	1742	52	50	264	2558	3320	1032	1165	1507	2762	1740
2005	7946	4009	3937	405	7526	5994	1810	64	78	306	2735	3705	1200	1252	1735	3017	1942
2006	8445	4313	4132	532	7886	6392	1893	91	69	299	2949	3899	1298	1338	1751	3297	2059
2007	9186	4811	4375	605	8563	6985	2027	76	98	341	3232	4302	1311	1574	1851	3449	2312
2008	10032	5250	4782	630	9366	7638	2177	91	126	367	3511	4708	1446	1690	2155	3745	2442
2009	11223	6031	5192	824	10332	8538	2447	140	98	427	3936	5352	1508	1928	2245	4441	2609
2010	11954	6491	5463	848	11052	9113	2575	138	128	433	4045	5817	1659	2134	2468	4579	2773
2011	13341	7246	6095	980	12307	10287	2775	133	146	479	4464	6506	1892	2293	2913	4965	3170
2012	14746	8066	6680	1073	13600	11354	3077	170	145	515	4720	7382	2129	2535	3164	5665	3382
2013	15741	8654	7087	1233	14439	12171	3257	176	137	497	4945	8013	2286	2613	3477	6134	3517
2014	17199	9617	7582	1351	15754	13264	3530	215	190	586	5306	8786	2521	2814	3903	6739	3743
2015	18702	10518	8184	1458	17127	14365	3901	190	246	624	5605	9761	2712	3117	4238	7239	4108
2016	19867	11136	8731	1599	18153	15228	4178	247	214	629	5976	10424	2838	3243	4404	7807	4413
2017	20993	12007	8986	1722	19148	16254	4238	239	262	689	6026	11172	3106	3392	4597	8294	4710
2018	22515	13064	9451	1798	20609	17427	4532	275	281	720	6140	12037	3582	3560	4978	8961	5016
2019	24117	13993	10124	2003	22021	18594	4911	309	303	767	6352	12974	4024	3724	5269	9991	5133
2020	37409	21202	16207	4601	32704	28009	8319	541	540	1109	9444	20248	6608	5531	8154	15910	7814
Total	294854	161536	133318	23297	270271	225088	63200	3307	3259	9932	90591	149211	45120	47952	64046	116071	66785

**Figure 1:**
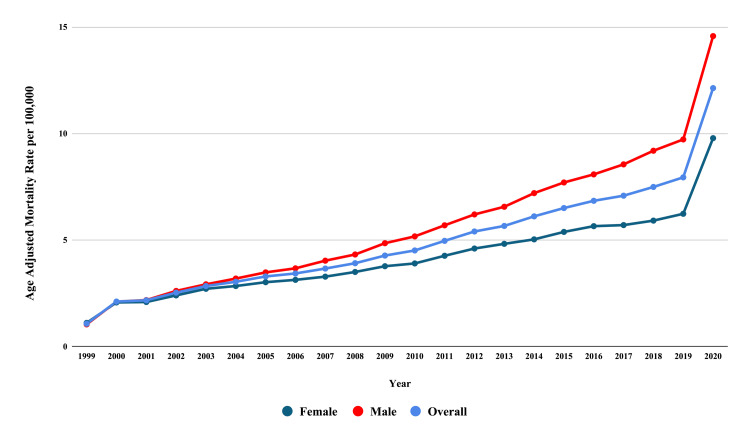
Age-Adjusted Mortality Rates per 100000 Population in the United States From 1999 Through 2020, Overall and Stratified by Sex.

The AAMR increased from 1.08 (95% CI: 1.04-1.13) in 1999 to 12.14 (95% CI: 12.01-12.26) in 2020 (Figure 1, Table 2, and Table 3). There has been a steady rise in AAMR since 1999, with a sudden spike after 2018. This spike in AAMR from 2019 was consistent among most groups. The cumulative AAPC for our cohort was +8.59 (95% CI: 7.00-10.2) (Table 3). The 2018-2020 APC reflects a short-term deviation from the longer pre-pandemic trend. The excess mortality analysis, which projects the expected 2020 AAMR based on the 1999-2019 pre-pandemic trajectory, provides a more appropriate framework for evaluating the impact of the COVID-19 pandemic on mortality in this population.

**Table 2:** Overall and Sex-Stratified Age-Adjusted Mortality Rates per 100000 in Obese Individuals With Hypertensive Diseases in the United States (1999-2020). CI: confidence interval

Year	Female (95% CI)	Male (95% CI)	Overall (95% CI)
1999	1.12 (1.05-1.18)	1.05 (0.98-1.11)	1.08 (1.04-1.13)
2000	2.08 (2.00-2.16)	2.12 (2.03-2.21)	2.12 (2.06-2.18)
2001	2.10 (2.02-2.18)	2.19 (2.10-2.28)	2.18 (2.11-2.24)
2002	2.41 (2.32-2.50)	2.62 (2.52-2.72)	2.54 (2.48-2.61)
2003	2.72 (2.63-2.81)	2.93 (2.83-3.04)	2.85 (2.78-2.92)
2004	2.85 (2.76-2.95)	3.20 (3.10-3.31)	3.05 (2.98-3.12)
2005	3.03 (2.94-3.13)	3.49 (3.38-3.60)	3.30 (3.22-3.37)
2006	3.14 (3.04-3.24)	3.68 (3.57-3.80)	3.44 (3.36-3.51)
2007	3.29 (3.19-3.38)	4.04 (3.93-4.16)	3.67 (3.59-3.75)
2008	3.51 (3.41-3.61)	4.33 (4.21-4.45)	3.92 (3.85-4.00)
2009	3.78 (3.68-3.89)	4.86 (4.74-4.98)	4.28 (4.20-4.36)
2010	3.91 (3.80-4.01)	5.18 (5.05-5.30)	4.52 (4.44-4.60)
2011	4.27 (4.16-4.38)	5.70 (5.57-5.83)	4.97 (4.89-5.06)
2012	4.61 (4.50-4.73)	6.21 (6.07-6.35)	5.41 (5.32-5.50)
2013	4.83 (4.72-4.95)	6.57 (6.42-6.71)	5.67 (5.58-5.76)
2014	5.04 (4.93-5.16)	7.21 (7.07-7.36)	6.12 (6.02-6.21)
2015	5.39 (5.27-5.51)	7.71 (7.55-7.86)	6.51 (6.42-6.61)
2016	5.66 (5.54-5.78)	8.09 (7.93-8.24)	6.85 (6.75-6.95)
2017	5.71 (5.59-5.83)	8.56 (8.40-8.71)	7.09 (6.99-7.19)
2018	5.92 (5.80-6.04)	9.20 (9.04-9.36)	7.50 (7.40-7.60)
2019	6.24 (6.12-6.37)	9.73 (9.57-9.90)	7.95 (7.85-8.06)
2020	9.79 (9.63-9.95)	14.58 (14.38-14.78)	12.14 (12.01-12.26)
Total	4.32 (4.30-4.34)	5.92 (5.89-5.95)	5.11 (5.09-5.13)

**Table 3:** Annual Percent Change (APC) and Average Annual Percent Change (AAPC) of Age-Adjusted Mortality Rates per 100000 in Obese Individuals With Hypertensive Diseases (1999-2020). CI: confidence interval

Year interval	APC (95% CI)	AAPC (95% CI)
Overall		
1999-2018	7.31 (6.43-8.20)	8.60 (7.00-10.22)
2018-2020	21.65 (4.92-41.05)	
Males		
1999-2001	47.04 (25.94-71.68)	12.34 (10.76-13.95)
2001-2018	7.78 (7.46-8.11)	
Female		
1999-2018	6.08 (5.29-6.88)	7.47 (5.89-9.07)
2018-2020	21.54 (4.49-41.38)	
15-34 years old		
1999-2002	47.57 (22.34-77.99)	12.24 (9.20-15.37)
2002-2018	5.81 (5.02-6.62)	
2018-2020	19.35 (4.80-35.92)	
35-54 years old		
1999-2001	36.17 (22.00-52.02)	10.80 (9.54-12.07)
2001-2015	7.88 (7.56-8.21)	
2015-2018	1.31 (-2.95-5.76)	
2018-2020	24.27 (19.68-29.04)	
55-74 years old		
1999-2018	6.90 (6.14-7.67)	8.34 (7.01-9.69)
2018-2020	23.04 (8.89-39.05)	
75+ years old		
1999-2018	7.39 (6.61-8.18)	9.35 (7.92-10.80)
2018-2020	29.88 (13.83-48.18)	
Hispanic		
1999-2018	6.64 (5.74-7.55)	9.97 (8.54-11.43)
2018-2020	47.29 (30.03-66.84)	
Non Hispanics		
1999-2001	39.84 (27.24-53.69)	10.94 (9.94-11.60)
2001-2018	6.92 (6.67-7.17)	
2018-2020	20.46 (15.84-25.26)	
American Indian or Alaska Native		
2000-2018	7.24 (5.66-8.84)	9.55 (7.04-12.13)
2018-2020	32.82 (7.41-64.23)	
Asian or Pacific Islander		
2000-2020	9.05 (7.19-10.94)	9.05 (7.19-10.94)
Black or African American		
1999-2018	5.44 (4.67-6.22)	7.37 (5.87-8.90)
2018-2020	27.57 (10.58-47.18)	
White		
1999-2001	42.27 (23.54-63.84)	11.44 (9.96-12.93)
2001-2018	7.26 (6.94-7.59)	
2018-2020	20.77 (14.55-27.32)	
Census Region 1: Northeast		
1999-2011	9.92 (8.39-11.48)	9.19 (7.51-10.91)
2011-2018	4.55 (1.88-7.30)	
2018-2020	22.08 (7.34-38.86)	
Census Region 2: Midwest		
1999-2020	8.83 (7.92-9.75)	8.83 (7.92-9.75)
Census Region 3: South		
1999-2018	7.25 (6.47-8.05)	8.89 (7.43-10.38)
2018-2020	25.75 (9.75-44.07)	
Census Region 4: West		
1999-2003	18.73 (11.40-26.55)	8.90 (7.45-10.38)
2003-2018	4.99 (4.43-5.55)	
2018-2020	20.60 (11.54-30.40)	
Urban		
1999-2018	7.29 (6.40-8.18)	8.53 (6.69-10.40)
2018-2020	21.12 (1.52-44.51)	
Rural		
1999-2001	47.95 (26.21-73.45)	12.24 (10.60-13.90)
2001-2018	7.45 (7.12-7.80)	
2018-2020	23.28 (17.07-29.82)	

Data for the place of death were available for 294237 of these deaths, which showed that 44.99% occurred at home, 42.65% of the deaths occurred in medical facilities, 5.65% occurred at nursing homes/long-term care facilities, and 0.94% occurred at hospice (Table 4).

**Table 4:** Mortality in Obese Individuals With Hypertensive Diseases, Stratified by the Place of Death in the United States (1999-2020).

Place of Death	Deaths
Medical facilities	125492
Decedent's home	132383
Hospice facility	2757
Nursing home/long-term care	16645
Others	16960
Place of death unknown	617
Total	294854

In our study group, a significant portion of the total deaths, 161536 (54.79%), occurred in men. The Southern US regions accounted for 116072 (39.37%) of the total deaths. The non-Hispanic population contributed to an overwhelming 270271 (91.66%) deaths. Within this group, 225088 (76.34%) deaths were among White individuals, while Black individuals accounted for 63200 (22.43%) deaths. About 2.23% of the deaths were attributed to Indian/Alaska Native and Asian/Pacific Islander populations.

The age distribution revealed that 149211 (50.60%) deaths were recorded in the 55-74-year-old age group, followed by 90591 (30.72%) deaths in the 35-54-year-old age group and 45120 (15.30%) in the 75+ age group. The 15-34-year-old age group just accounted for 9932 (3.36%) deaths (Table 1).

Obesity and hypertension AAMR stratified by sex

Over the study period, men aged ≥15 years had a higher AAMR than women aged ≥15 years (overall AAMR: men = 5.92; 95% CI, 5.85-5.95; women = 4.32; 95% CI: 4.30-4.34). The AAPC was significantly higher in men (AAPC: +12.34%; 95% CI, 10.75-13.94) than in women (AAPC: +7.46%; 95% CI, 5.89-9.07) (Figure 1, Table 2, and Table 3). 

Obesity and hypertension AAMR stratified by age groups

Mortality was highest among the 55-74-year-old age group, followed by 75-plus-year-old, 35-54-year-old, and finally 15-34-year-old age group (AAMR: 55-74: 11.76; 95% CI, 11.70-11.82; 75+: 10.83; 95% CI, 10.73-10.93; 35-54: 4.73; 95% CI, 4.70-4.76; and 15-34: 0.54; 95% CI, 0.53-0.56).

**Figure 2:**
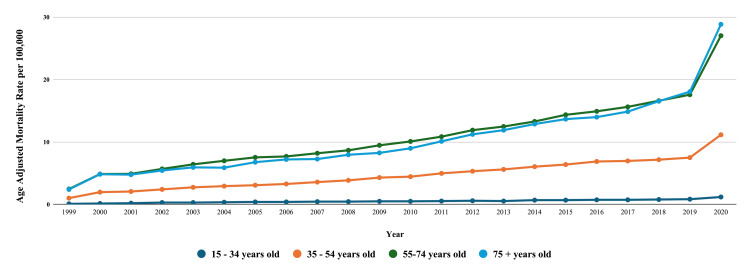
Age-Adjusted Mortality Rates per 100000 Population From 1999 to 2020, Stratified by Age Group. Mortality trends are shown for individuals aged 15-34 years (dark blue), 35-54 years (orange), 55-74 years (green), and 75 years and older (light blue).

However, throughout the study, the younger age groups showed the highest overall rate of increase in AAMRs. The highest was demonstrated by the 15-34-year-old age group (AAPC: +12.24; 95% CI, 9.20-15.37); they were followed by 35-54 year olds (AAPC: +10.80; 95% CI, 9.54-12.07) and 75+ year olds (AAPC: +9.35; 95% CI, 7.92-10.80), and 55-74 year olds actually showed the lowest overall increase in AAMR (AAPC: +8.34; 95% CI, 7.01-9.69) (Figure 2, Table 3, and Table 5).

**Table 5:** Age-Adjusted Mortality Rates per 100000 in Obese Individuals With Hypertensive Diseases in the United States According to Age Groups (1999-2020). CI: confidence interval

Year	15-34 Years Old (95% CI)	35-54 Years Old (95% CI)	55-74 Years Old (95% CI)	75+ Years Old (95% CI)
1999	0.10 (0.08-0.12)	1.01 (0.94-1.08)	2.47 (2.32-2.62)	2.40 (2.16-2.64)
2000	0.15 (0.12-0.17)	1.97 (1.88-2.07)	4.87 (4.66-5.08)	4.84 (4.51-5.18)
2001	0.20 (0.17-0.23)	2.07 (1.97-2.17)	4.90 (4.69-5.11)	4.77 (4.44-5.09)
2002	0.30 (0.26-0.34)	2.41 (2.30-2.51)	5.69 (5.47-5.91)	5.44 (5.09-5.79)
2003	0.30 (0.26-0.34)	2.74 (2.63-2.85)	6.42 (6.18-6.65)	5.94 (5.58-6.31)
2004	0.35 (0.31-0.39)	2.93 (2.82-3.05)	6.99 (6.75-7.23)	5.89 (5.53-6.25)
2005	0.40 (0.35-0.44)	3.08 (2.96-3.19)	7.54 (7.30-7.79)	6.77 (6.38-7.15)
2006	0.40 (0.35-0.44)	3.28 (3.16-3.40)	7.69 (7.44-7.93)	7.21 (6.82-7.60)
2007	0.45 (0.40-0.49)	3.58 (3.45-3.70)	8.20 (7.95-8.45)	7.27 (6.87-7.66)
2008	0.45 (0.40-0.49)	3.86 (3.73-3.99)	8.67 (8.42-8.92)	7.96 (7.55-8.37)
2009	0.50 (0.45-0.54)	4.30 (4.16-4.43)	9.47 (9.22-9.73)	8.26 (7.85-8.68)
2010	0.50 (0.45-0.54)	4.45 (4.31-4.59)	10.09 (9.83-10.35)	8.99 (8.55-9.42)
2011	0.54 (0.50-0.59)	4.98 (4.83-5.12)	10.86 (10.59-11.13)	10.11 (9.65-10.57)
2012	0.59 (0.54-0.65)	5.32 (5.17-5.48)	11.90 (11.63-12.18)	11.23 (10.75-11.71)
2013	0.54 (0.50-0.59)	5.61 (5.46-5.77)	12.48 (12.20-12.75)	11.90 (11.41-12.39)
2014	0.69 (0.64-0.75)	6.05 (5.89-6.21)	13.29 (13.01-13.57)	12.88 (12.37-13.38)
2015	0.69 (0.64-0.75)	6.39 (6.22-6.55)	14.36 (14.08-14.65)	13.67 (13.15-14.19)
2016	0.74 (0.69-0.80)	6.88 (6.70-7.05)	14.92 (14.63-15.21)	13.99 (13.47-14.51)
2017	0.74 (0.69-0.80)	6.97 (6.79-7.14)	15.63 (15.34-15.92)	14.87 (14.35-15.40)
2018	0.79 (0.74-0.85)	7.17 (6.99-7.35)	16.61 (16.31-16.90)	16.54 (16.00-17.08)
2019	0.84 (0.78-0.90)	7.50 (7.32-7.69)	17.58 (17.27-17.88)	18.06 (17.50-18.62)
2020	1.19 (1.12-1.26)	11.16 (10.94-11.39)	27.03 (26.66-27.40)	28.85 (28.15-29.54)
Total	0.54 (0.53-0.56)	4.73 (4.70-4.76)	11.76 (11.70-11.82)	10.83 (10.73-10.93)

Obesity and hypertension AAMR stratified by race and ethnicity

Mortality was higher in non-Hispanic populations as compared to Hispanic populations (AAMR: non-Hispanic = 5.22; 95% CI, 5.20-5.24; Hispanic = 4.08; 95% CI, 4.03-4.14). AAPCs were comparable in non-Hispanic (AAPC: +10.94; 95% CI, 9.94-11.95) and Hispanic populations (AAPC: +9.97; 95% CI, 8.53-11.42).

Among the non-Hispanic (NH) races, NH Black or African American had the highest overall AAMR (9.81; 95% CI, 9.74-9.89), followed by NH American Indian or Alaska Native (6.01; 95% CI, 5.80-6.23), NH White (4.64; 95% CI, 4.62-4.66), and finally NH Asian or Pacific Islander (1.17; 95% CI, 1.13-1.21).

**Figure 3:**
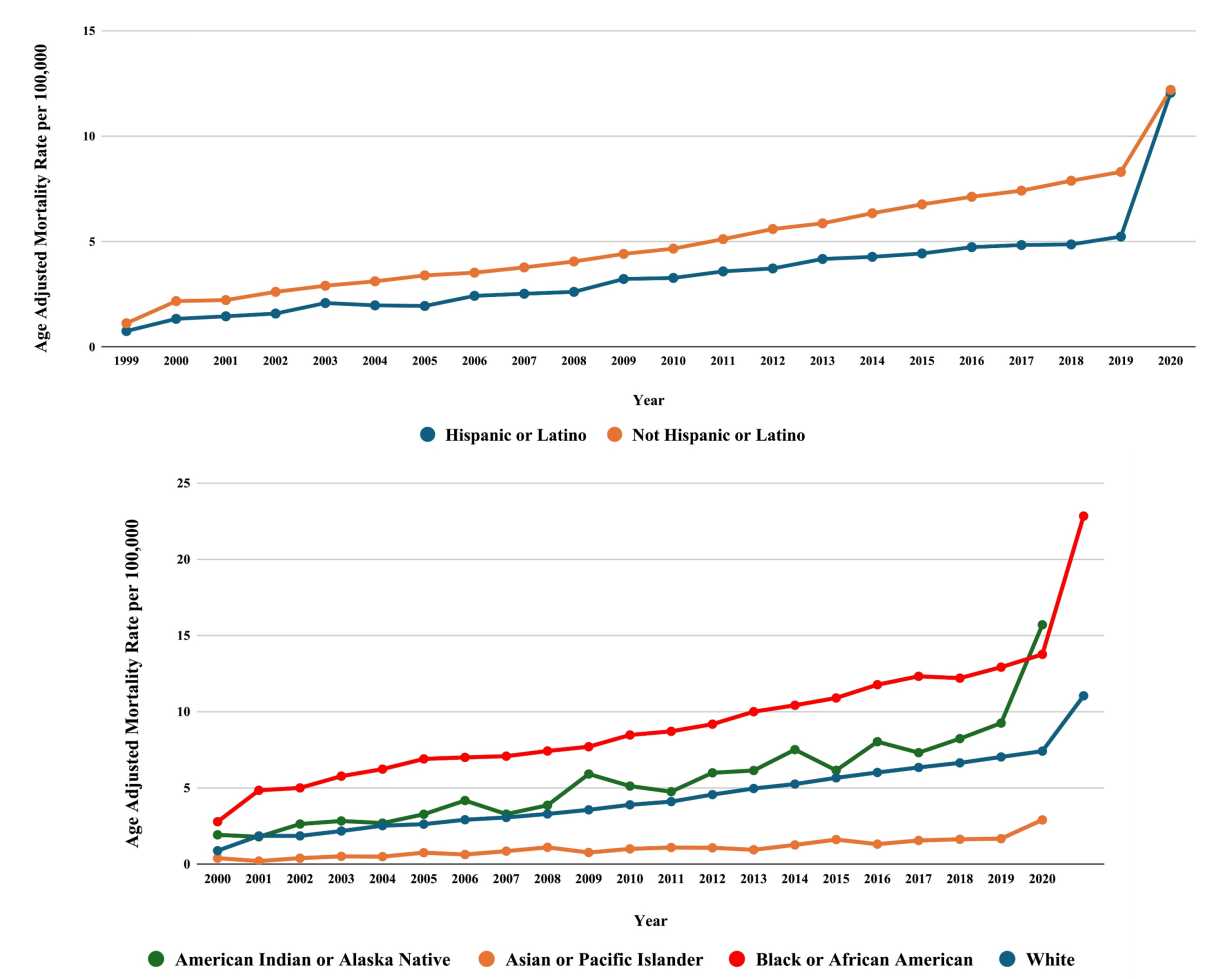
Age-Adjusted Mortality Rates per 100000 Population From 1999 to 2020, Stratified by Ethnicity (Top Panel) and Race (Bottom Panel). The top panel shows Hispanic or Latino (blue) and not Hispanic or Latino (orange). The bottom panel shows American Indian or Alaska Native (green), Asian or Pacific Islander (orange), Black or African American (red), and White (blue).

However, the highest overall increase in AAMR throughout the study represented by AAPC was shown by NH White (AAPC: +11.44; 95% CI, 9.64-12.93); they were followed by NH American Indian or Alaska Native and NH Asian or Pacific Islander, which both showed similar values (AAPC: +9.56; 95% CI, 7.05-12.13; AAPC: +9.05; 95% CI, 7.19-10.94 respectively). Finally, NH Black or African American showed the lowest AAPC (+7.37; 95% CI, 5.87-8.90) (Figure 3, Table 3, and Table 6).

**Table 6:** Age-Adjusted Mortality Rates per 100000 in Obese Individuals With Hypertensive Diseases in the United States According to Race and Ethnicity (1999 to 2020). CI: confidence interval

Year	American Indian or Alaska Native (95% CI)	Asian or Pacific Islander (95% CI)	Black or African American (95% CI)	White (95% CI)	Hispanic or Latino (95% CI)	Not Hispanic or Latino (95% CI)
1999	Unreliable	Unreliable	2.80 (2.58-3.03)	0.90 (0.86-0.94)	0.77 (0.62-0.92)	1.14 (1.09-1.18)
2000	1.94 (1.24-2.88)	0.41 (0.27-0.58)	4.86 (4.57-5.15)	1.87 (1.81-1.94)	1.35 (1.15-1.54)	2.19 (2.13-2.26)
2001	1.81 (1.21-2.60)	0.22 (0.13-0.35)	5.02 (4.73-5.31)	1.87 (1.81-1.94)	1.47 (1.28-1.67)	2.24 (2.17-2.30)
2002	2.65 (1.84-3.68)	0.41 (0.29-0.58)	5.79 (5.48-6.10)	2.19 (2.12-2.26)	1.60 (1.41-1.80)	2.63 (2.56-2.70)
2003	2.85 (2.04-3.86)	0.53 (0.39-0.72)	6.25 (5.93-6.57)	2.54 (2.47-2.61)	2.10 (1.87-2.32)	2.92 (2.85-3.00)
2004	2.71 (1.97-3.63)	0.51 (0.38-0.68)	6.92 (6.58-7.25)	2.64 (2.57-2.71)	1.99 (1.78-2.19)	3.13 (3.06-3.21)
2005	3.29 (2.49-4.26)	0.77 (0.60-0.97)	7.02 (6.69-7.35)	2.93 (2.86-3.00)	1.96 (1.76-2.17)	3.41 (3.33-3.48)
2006	4.19 (3.32-5.21)	0.65 (0.50-0.83)	7.10 (6.77-7.43)	3.08 (3.00-3.16)	2.44 (2.22-2.66)	3.54 (3.46-3.62)
2007	3.30 (2.55-4.20)	0.87 (0.71-1.07)	7.44 (7.11-7.77)	3.31 (3.23-3.38)	2.54 (2.33-2.76)	3.79 (3.71-3.87)
2008	3.88 (3.08-4.83)	1.12 (0.91-1.32)	7.72 (7.39-8.05)	3.58 (3.50-3.66)	2.63 (2.41-2.85)	4.07 (3.99-4.16)
2009	5.93 (4.86-6.99)	0.78 (0.63-0.96)	8.49 (8.15-8.84)	3.91 (3.83-3.99)	3.24 (3.01-3.48)	4.43 (4.34-4.52)
2010	5.14 (4.23-6.04)	1.02 (0.84-1.21)	8.73 (8.38-9.07)	4.12 (4.04-4.21)	3.29 (3.05-3.52)	4.68 (4.59-4.76)
2011	4.77 (3.91-5.62)	1.11 (0.93-1.30)	9.20 (8.85-9.55)	4.58 (4.49-4.67)	3.60 (3.36-3.84)	5.13 (5.04-5.22)
2012	6.01 (5.06-6.96)	1.09 (0.91-1.27)	10.02 (9.65-10.38)	4.98 (4.89-5.07)	3.74 (3.51-3.98)	5.61 (5.51-5.71)
2013	6.16 (5.21-7.11)	0.96 (0.80-1.13)	10.44 (10.07-10.80)	5.27 (5.17-5.36)	4.19 (3.94-4.43)	5.88 (5.78-5.98)
2014	7.53 (6.47-8.59)	1.28 (1.09-1.46)	10.92 (10.55-11.29)	5.68 (5.58-5.78)	4.29 (4.05-4.53)	6.36 (6.26-6.46)
2015	6.17 (5.26-7.08)	1.63 (1.43-1.84)	11.79 (11.41-12.17)	6.03 (5.93-6.13)	4.45 (4.21-4.69)	6.78 (6.68-6.89)
2016	8.05 (7.01-9.09)	1.33 (1.15-1.51)	12.34 (11.96-12.72)	6.36 (6.25-6.46)	4.75 (4.51-5.00)	7.14 (7.03-7.25)
2017	7.33 (6.37-8.29)	1.57 (1.38-1.76)	12.22 (11.85-12.60)	6.66 (6.55-6.77)	4.85 (4.61-5.08)	7.43 (7.32-7.54)
2018	8.25 (7.25-9.25)	1.65 (1.45-1.84)	12.94 (12.56-13.33)	7.05 (6.94-7.15)	4.88 (4.65-5.11)	7.90 (7.79-8.01)
2019	9.27 (8.21-10.34)	1.69 (1.50-1.89)	13.78 (13.38-14.17)	7.43 (7.32-7.54)	5.25 (5.01-5.49)	8.32 (8.20-8.43)
2020	15.72 (14.36-17.08)	2.92 (2.67-3.17)	22.85 (22.35-23.36)	11.06 (10.92-11.19)	12.07 (11.71-12.43)	12.21 (12.07-12.35)
Total	6.01 (5.80-6.23)	1.17 (1.13-1.21)	9.81 (9.74-9.89)	4.64 (4.62-4.66)	4.08 (4.03-4.14)	5.22 (5.20-5.24)

Obesity and hypertension AAMR stratified by geographic region

Inspecting the trends, a substantial difference was observed in different states. When arranged by AAMR, states that fell in the top 90th percentile included Vermont, Oklahoma, the District of Columbia, West Virginia, Mississippi, and Rhode Island. States that fell into the bottom 10th percentile were Connecticut, Virginia, Massachusetts, Missouri, Maine, and Nebraska. Vermont exhibited the highest overall AAMR at 12.44 (95% CI: 11.83-13.05), more than four times that of Connecticut, with the lowest AAMR at 2.67 (95% CI: 2.55-2.8) (Table 7).

**Table 7:** Age-Adjusted Mortality Rates per 100000 in Obese Individuals With Hypertensive Diseases in the United States, by State (1999-2020). CI: confidence interval

State	Age Adjusted Rate (95% CI)
Alabama	2.78 (2.67-2.89)
Alaska	5.99 (5.50-6.48)
Arizona	4.38 (4.26-4.50)
Arkansas	4.44 (4.26-4.62)
California	5.33 (5.28-5.39)
Colorado	6.31 (6.15-6.48)
Connecticut	2.67 (2.55-2.80)
Delaware	7.07 (6.67-7.47)
District of Columbia	9.62 (9.02-10.21)
Florida	4.45 (4.38-4.51)
Georgia	5.28 (5.17-5.40)
Hawaii	4.23 (3.98-4.49)
Idaho	4.59 (4.33-4.84)
Illinois	3.9 (3.82-3.98)
Indiana	4.95 (4.82-5.07)
Iowa	5.9 (5.71-6.10)
Kansas	4.25 (4.07-4.42)
Kentucky	5.52 (5.36-5.68)
Louisiana	6.19 (6.02-6.37)
Maine	3.8 (3.57-4.03)
Maryland	4.82 (4.68-4.95)
Massachusetts	2.93 (2.84-3.03)
Michigan	5.31 (5.21-5.41)
Minnesota	5.88 (5.73-6.03)
Mississippi	7.14 (6.91-7.37)
Missouri	3.67 (3.56-3.79)
Montana	5.36 (5.03-5.68)
Nebraska	3.75 (3.55-3.96)
Nevada	5.45 (5.24-5.66)
New Hampshire	4.05 (3.81-4.30)
New Jersey	3.86 (3.76-3.95)
New Mexico	5.55 (5.31-5.79)
New York	5.31 (5.23-5.38)
North Carolina	5.67 (5.56-5.79)
North Dakota	5.81 (5.40-6.23)
Ohio	5.55 (5.45-5.65)
Oklahoma	10.87 (10.63-11.12)
Oregon	5.08 (4.92-5.24)
Pennsylvania	4.1 (4.02-4.18)
Rhode Island	6.86 (6.50-7.22)
South Carolina	5.35 (5.20-5.51)
South Dakota	4.32 (3.99-4.65)
Tennessee	6.46 (6.32-6.61)
Texas	6.46 (6.38-6.54)
Utah	4.05 (3.85-4.25)
Vermont	12.44 (11.83-13.05)
Virginia	2.86 (2.77-2.94)
Washington	4.7 (4.58-4.82)
West Virginia	7.08 (6.80-7.36)
Wisconsin	6.74 (6.59-6.90)
Wyoming	6.53 (6.04-7.03)

**Figure 4:**
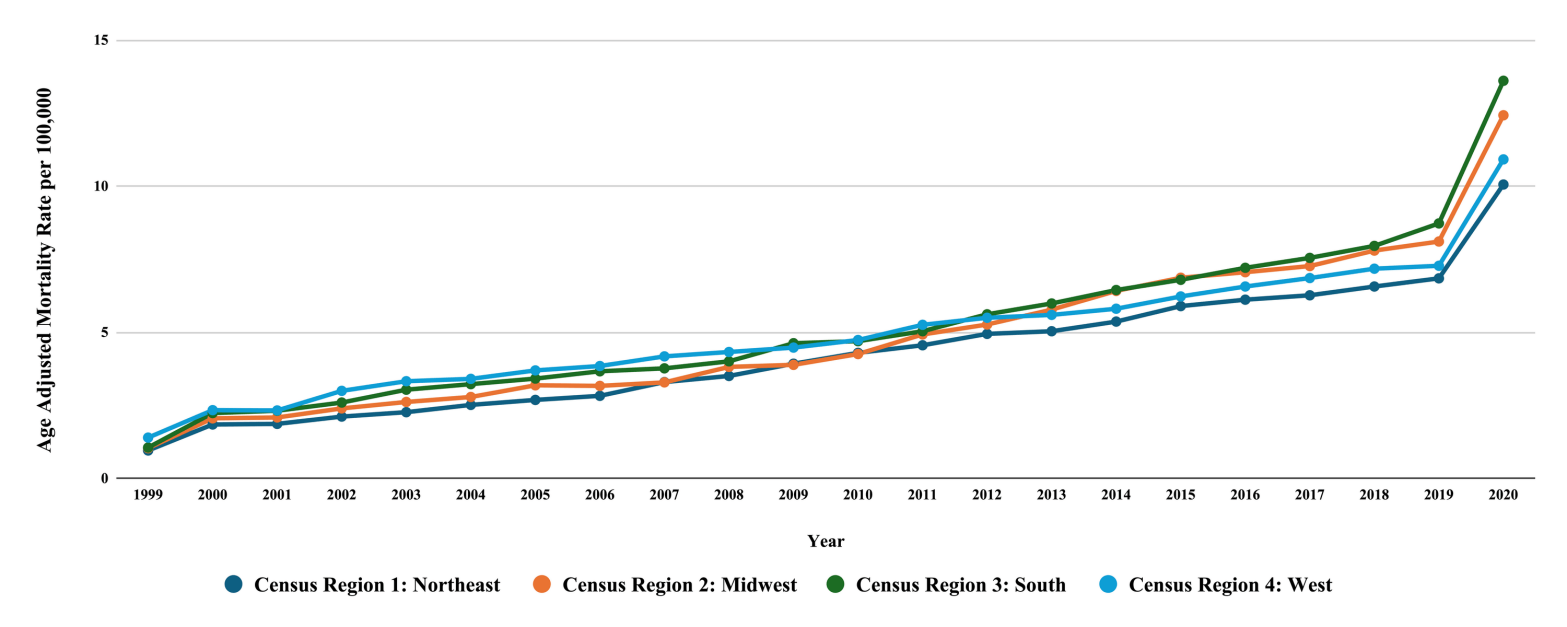
Age-Adjusted Mortality Rates per 100000 Population From 1999 to 2020, Stratified by US Census Region. Trends are shown for the Northeast (dark blue), Midwest (orange), South (green), and West (light blue).

During the study period between 1999 and 2020, overall AAMR was highest in the Southern US region, followed by the Western, Midwestern, and Northeast US regions. In terms of AAPC values, all four regions exhibited similar values; however, the Northeast region showed the highest AAPC (Northeast AAPC: +9.19; 95% CI, 7.51-10.90; West AAPC: +8.90; 95% CI, 7.45-10.38; South AAPC: +8.89; 95% CI, 7.42-10.38; and Midwest AAPC: +8.83; 95% CI, 7.92-9.75) (Figure 4, Table 3, and Table 8).

**Table 8:** Age-Adjusted Mortality Rates per 100000 in Obese Individuals With Hypertensive Diseases in the United States According to Census Regions (1999-2020). CI: confidence interval

Year	Census Region 1: Northeast (95% CI)	Census Region 2: Midwest (95% CI)	Census Region 3: South (95% CI)	Census Region 4: West (95% CI)
1999	0.96 (0.86-1.05)	1.04 (0.95-1.13)	1.06 (0.99-1.14)	1.40 (1.29-1.51)
2000	1.85 (1.72-1.97)	2.06 (1.94-2.19)	2.24 (2.14-2.35)	2.34 (2.20-2.48)
2001	1.87 (1.74-2.00)	2.09 (1.97-2.22)	2.32 (2.21-2.43)	2.33 (2.20-2.47)
2002	2.12 (1.98-2.25)	2.40 (2.27-2.53)	2.60 (2.49-2.71)	3.00 (2.84-3.15)
2003	2.27 (2.13-2.41)	2.62 (2.48-2.76)	3.04 (2.92-3.16)	3.33 (3.16-3.49)
2004	2.52 (2.38-2.67)	2.79 (2.65-2.93)	3.23 (3.10-3.35)	3.41 (3.25-3.57)
2005	2.69 (2.54-2.84)	3.19 (3.04-3.34)	3.42 (3.30-3.55)	3.70 (3.54-3.87)
2006	2.83 (2.67-2.98)	3.17 (3.02-3.32)	3.67 (3.54-3.80)	3.85 (3.69-4.02)
2007	3.30 (3.13-3.46)	3.29 (3.14-3.44)	3.77 (3.65-3.90)	4.18 (4.01-4.35)
2008	3.51 (3.34-3.68)	3.82 (3.65-3.98)	4.01 (3.88-4.14)	4.33 (4.16-4.51)
2009	3.93 (3.75-4.10)	3.89 (3.73-4.05)	4.63 (4.49-4.77)	4.48 (4.31-4.66)
2010	4.30 (4.11-4.48)	4.26 (4.09-4.43)	4.70 (4.57-4.84)	4.74 (4.56-4.92)
2011	4.56 (4.37-4.75)	4.93 (4.75-5.11)	5.04 (4.90-5.19)	5.26 (5.07-5.44)
2012	4.95 (4.76-5.15)	5.27 (5.09-5.46)	5.62 (5.47-5.77)	5.50 (5.32-5.69)
2013	5.04 (4.84-5.24)	5.78 (5.58-5.97)	5.99 (5.84-6.14)	5.60 (5.41-5.78)
2014	5.37 (5.17-5.58)	6.42 (6.22-6.63)	6.45 (6.29-6.61)	5.81 (5.62-6.00)
2015	5.90 (5.68-6.11)	6.87 (6.66-7.09)	6.80 (6.64-6.96)	6.23 (6.03-6.42)
2016	6.12 (5.90-6.34)	7.06 (6.85-7.28)	7.21 (7.05-7.37)	6.57 (6.38-6.77)
2017	6.27 (6.06-6.49)	7.27 (7.05-7.48)	7.55 (7.38-7.72)	6.86 (6.66-7.06)
2018	6.57 (6.35-6.80)	7.80 (7.57-8.02)	7.96 (7.79-8.13)	7.18 (6.98-7.39)
2019	6.85 (6.62-7.08)	8.11 (7.89-8.34)	8.73 (8.55-8.91)	7.28 (7.08-7.49)
2020	10.06 (9.78-10.33)	12.43 (12.15-12.71)	13.61 (13.39-13.83)	10.92 (10.67-11.17)
Total	4.37 (4.33-4.41)	5.02 (4.98-5.06)	5.47 (5.43-5.50)	5.19 (5.15-5.22)

**Figure 5:**
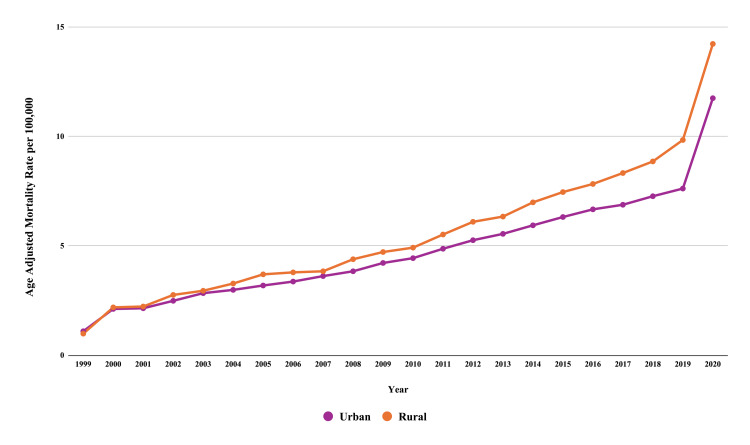
Age-Adjusted Mortality Rates per 100000 Population From 1999 to 2020, Stratified by Urbanity. Trends are shown for urban (purple) and rural (orange) areas.

Non-metropolitan areas (rural areas) consistently had higher obesity- and hypertension-related AAMRs (5.63; 95% CI, 5.58-5.68) than metropolitan areas (urban areas) (4.99; 95% CI, 4.97-5.01). Rural areas also showed the highest increase in AAMR (AAPC: +12.24; 95% CI, 10.60-13.90). Urban areas show an AAPC of +8.53 (95% CI: 6.69-10.40). Notably, rural areas showed a significant, steep increase in AAMR between 1999 and 2001 (APC: 47.95; 95% CI, 26.20-73.45) (Figure 5, Table 3, and Table 9).

**Table 9:** Age-Adjusted Mortality Rates per 100000 in Obese Individuals With Hypertensive Diseases in the United States According to Urban/Rural Classification (1999-2020). CI: confidence interval

Year	Urban (95% CI)	Rural (95% CI)
1999	1.10 (1.05-1.15)	0.99 (0.89-1.09)
2000	2.12 (2.05-2.19)	2.19 (2.05-2.34)
2001	2.15 (2.09-2.22)	2.23 (2.08-2.38)
2002	2.49 (2.42-2.56)	2.76 (2.60-2.93)
2003	2.84 (2.77-2.92)	2.95 (2.78-3.12)
2004	2.99 (2.92-3.07)	3.28 (3.10-3.46)
2005	3.19 (3.11-3.27)	3.70 (3.51-3.89)
2006	3.37 (3.29-3.45)	3.79 (3.60-3.97)
2007	3.62 (3.53-3.70)	3.84 (3.64-4.03)
2008	3.84 (3.75-3.92)	4.39 (4.18-4.59)
2009	4.22 (4.13-4.31)	4.72 (4.51-4.93)
2010	4.44 (4.35-4.53)	4.92 (4.70-5.13)
2011	4.87 (4.78-4.96)	5.52 (5.29-5.75)
2012	5.26 (5.17-5.36)	6.10 (5.86-6.34)
2013	5.55 (5.45-5.65)	6.34 (6.09-6.58)
2014	5.94 (5.84-6.04)	6.99 (6.74-7.25)
2015	6.32 (6.22-6.43)	7.46 (7.20-7.73)
2016	6.67 (6.57-6.78)	7.83 (7.56-8.11)
2017	6.88 (6.78-6.99)	8.33 (8.05-8.61)
2018	7.27 (7.16-7.38)	8.86 (8.57-9.15)
2019	7.62 (7.51-7.73)	9.84 (9.54-10.15)
2020	11.75 (11.62-11.89)	14.23 (13.87-14.59)
Total	4.99 (4.97-5.01)	5.63 (5.58-5.68)

Estimated excess mortality in 2020

In total, 10508 excess deaths occurred in 2020 (28.09% increase). These data were calculated using the AAPC from 1999 to 2019 to project the expected AAMR for 2020. Women showed 5016 excess deaths (30.95% increase). Men exhibited 5511 excess deaths (25.99% increase). In age groups, the 75+ age group showed the largest increase (2155 excess deaths, 32.62% increase). This was followed by 55-74 (6158 excess deaths, 30.41% increase), 35-54 (2496 excess deaths, 26.43% increase), and 15-34 (242 excess deaths, 21.85% increase). When stratifying by ethnicity and race, Hispanics showed 2447 excess deaths (53.19% increase), which was the highest increase shown by any demographic. Non-Hispanics showed 8223 deaths (25.14% increase). NH Asian or Pacific Islander, NH American Indian, and NH Black or African American all showed similar increases (37.67%, 36.58%, and 35.05%, respectively), with NH Whites showing the lowest percent increase at 25.86%. Furthermore, metro areas showed higher increases (8998 excess deaths, 28.94% increase) than non-metro areas (1680 excess deaths, 25.23% increase), with the South region showing the highest increase (4945 excess deaths, 31.08% increase) (Table 10).

**Table 10:** Estimated Excess Mortality in Obese Individuals With Hypertensive Diseases in the United States During the COVID-19 Pandemic (2020). AAPC, average annual percent change; CI, confidence interval; AAMR, age-adjusted mortality rate

Group	AAPC Until 2019 (95% CI)	Projected AAMR (2019-2020)	Actual AAMR (2020)	Estimated Excess AAMR	Percent Excess AAMR	Estimated Excess Deaths
Summary						
Overall	9.84 (8.28-11.42)	8.73	12.14	3.41	28.09	10508
Sex						
Male	10.94 (10.13-11.75)	10.79	14.58	3.79	25.99	5511
Female	8.34 (6.68-10.02)	6.76	9.79	3.03	30.95	5016
Ethnicity						
Hispanic	7.66 (6.55-8.79)	5.65	12.07	6.42	53.19	2447
Non-Hispanic	9.87 (8.57-11.19)	9.14	12.21	3.07	25.14	8223
Race						
Black or African American	7.72 (6.30-9.15)	14.84	22.85	8.01	35.05	2916
White	10.37 (8.53-12.24)	8.2	11.06	2.86	25.86	7243
American Indian	7.56 (6.35-8.79)	9.97	15.72	5.75	36.58	198
Asian or Pacific Islander	7.47 (5.92-9.04)	1.82	2.92	1.1	37.67	203
US census region						
Northeast	7.99 (6.95-9.04)	7.4	10.06	2.66	26.44	1462
Midwest	8.03 (7.37-8.70)	8.76	12.43	3.67	29.53	2407
South	7.42 (6.78-8.06)	9.38	13.61	4.23	31.08	4945
West	8.03 (6.36-9.72)	7.86	10.92	3.06	28.02	2190
Metro status						
Metro	9.60 (8.14-11.08)	8.35	11.75	3.4	28.94	8898
Non-metro	8.15 (7.39-8.92)	10.64	14.23	3.59	25.23	1680
Age group						
15-34	11.29 (8.59-14.07)	0.93	1.19	0.26	21.85	242
35-54	9.45 (8.36-10.55)	8.21	11.16	2.95	26.43	2496
55-74	7.02 (6.41-7.64)	18.81	27.03	8.22	30.41	6158
75+	7.64 (7.03-8.24)	19.44	28.85	9.41	32.62	2155

## Discussion

We conducted a comprehensive analysis of mortality data spanning two decades using the CDC WONDER database to identify cases involving both obesity and hypertension listed as the cause of death. We report several key findings from this investigation. Across all subgroups, AAMR rose steadily from 1999 to 2020, with a more than 11-fold increase over 21 years. Studies from the National Health and Nutrition Examination Survey (NHANES) show that the prevalence of hypertension and obesity during 2017-2020 was 44.7% and 41.9%, respectively [13,14]. Both these conditions have shown an increasing trend over the past two decades. Recent research shows that all-cause mortality was highest in obese individuals with uncontrolled hypertension, followed by those with controlled hypertension and, lastly, those with no hypertension [15].

Overall, men consistently showed a higher mortality rate than women. The 55-74-year-old cohort with obesity and hypertension had the highest risk of mortality as compared to the other age groups. When comparing ethnicities, the non-Hispanic population had higher mortality rates. Notably, non-Hispanic African Americans exhibited the highest mortality rates compared to other racial groups. Significant regional differences were also observed. Rural areas also showed higher AAMRs than urban areas. These results have important public health policy implications.

Our results also show that men have higher mortality rates compared to women. This difference is multifactorial and can be attributed to various factors; estrogen in premenopausal women is protective for cardiovascular health. It enhances endothelial function by increasing nitric oxide production, promotes vasodilation, and inhibits vascular smooth muscle proliferation. It also downregulates angiotensin-converting enzyme and angiotensin II receptors, reducing vasoconstriction and sodium retention, thereby lowering blood pressure and decreasing cardiovascular strain [16,17].

While absolute mortality rates remained substantially higher in the 55-74 age group throughout the study period, the younger age groups (15-34 and 35-54) showed the steepest increases, as indicated by their AAPC values. The observed mortality trends reflect a convergence of epidemiologic and social trends. Since the older adult US population carries a higher burden of obesity and hypertension, they see higher overall cumulative mortality rates [18]. Interestingly, between 1990 and 2021, the age-standardized percentage increase in obesity prevalence was substantially higher among adolescents aged 15-24 years (158.4% in men; 185.9% in women) compared to adults (123.6% in men; 99.9% in women) [19]. In addition to this, we are also seeing rising early-onset hypertension but lower awareness, treatment, and control among young adults aged 18-39 years compared to those ≥40 years [20]. While hypertension becomes more prevalent with advancing age, it remains a largely modifiable condition and is manageable with appropriate interventions. The concurrent and substantial rise in both obesity and hypertension among younger populations presents a more concerning trend. This compounded disease burden across age groups not only intensifies the overall strain on healthcare systems but also contributes to worsening outcomes, particularly among younger individuals who are now developing high-risk cardiometabolic conditions at earlier ages.

Our results showed that AAMRs were higher in non-Hispanic populations than in Hispanic populations. Among non-Hispanic groups, Black individuals exhibited the highest overall AAMRs, a trend consistent with prior research. According to the US National Health and Nutrition Examination Survey, 21% of hypertension cases in Black men and 18% in Black women can be attributed to being overweight. African American women and girls are more overweight and obese than those of other ethnic groups, making them more impacted by this correlation [21]. The increased rates of obesity in African Americans can be linked to the creation of food deserts in the United States, with African Americans being the predominant population. Studies have shown that socioeconomically vulnerable populations have poor access to healthy diets and higher rates of obesity and hypertension worldwide [2,22]. The highest overall increase in AAMR was in the non-Hispanic White population, with the lowest AAPC seen in the non-Hispanic black population. While Black or African American individuals still experience higher obesity rates overall, a study conducted from 1997 to 2008 revealed that BMI rose by 1 kg/m² or more across all race-sex groups. Notably, the White population exhibited a quicker increase during this period [23]. This information is valuable because it shows that several factors, such as sex, education, and socioeconomic status, affect the burden of these comorbidities on the US healthcare system.

Our study highlighted multiple geographic disparities. Of note are the steadily increasing mortality rates in rural areas compared to urban areas. This trend was observed consistently over 21 years. This difference can be attributed to a complex interplay of socioeconomic and behavioral factors unique to rural settings. One of the primary contributors is limited access to healthcare. According to the CDC, rural Americans are more likely to experience preventable hospitalizations and die prematurely from chronic diseases due to a lack of screening and regular follow-ups [24]. Moreover, the built environment in rural areas often promotes obesity. Unlike urban areas, rural communities often have fewer walkable destinations, limited recreational facilities, and less access to fresh food. Consequently, residents may depend on high-calorie, processed options from convenience stores, especially in "food deserts" with few supermarkets [25,26]. Overall, these interwoven factors create a uniquely high-risk environment in rural America, one that promotes obesity and undermines hypertension management, ultimately contributing to the persistent and growing disparity in obesity- and hypertension-related mortality as compared to metropolitan regions. The Northeast census region recorded the highest AAPC but the lowest AAMR, highlighting the critical need for heightened awareness among physicians in this region toward this patient population. Our results essentially signify that factors beyond raw disease prevalence drive rising mortality among people with multiple cardiometabolic conditions in certain areas.

A notable finding from our study was the sharp increase in AAMR for deaths related to obesity and hypertension, which nearly doubled from 1.08 in 1999 to 2.12 in 2000 (Table 2). This steep rise can be attributed to a reporting artifact during those years. In 1999, the United States transitioned from ICD-9 to ICD-10 for recording causes of death on death certificates. Previously, deaths were classified under broader categories such as heart disease or stroke. However, with the adoption of the ICD-10 codes, causes of death began to be recorded more specifically, such as "hypertensive heart disease" or "obesity-related complications." As a result, this sharp increase likely reflects an improved accuracy in capturing obesity- and hypertension-related deaths in the records, rather than a true, sudden doubling of the disease burden.

Our results exhibited a 28% increase in mortality during the early years of the COVID-19 pandemic. Hypertension combined with obesity is a risk factor for mortality from COVID-19, so much so that Perez et al. termed it a "collision of pandemics" [27]. Substantial evidence supports that increasing magnitudes of obesity are associated with an increased risk of mechanical ventilation, which contributes to a drastically increased risk of mortality from COVID-19 [28]. Lockdown protocols in the early pandemic years also proved a double-edged sword, as the limiting of daily physical activities led to a sedentary lifestyle and the increased prevalence of obesity; however, at the same time, obese individuals are at an increased risk of severe COVID-19 infection [29]. Although obesity and hypertension directly increased the risk of mortality in patients with COVID-19, the pandemic also led to a sharp decline in cardiovascular outpatient care, which may have contributed to additional non-COVID-19-related mortality [30].

Our study also showed a disproportionate effect on Hispanic populations, which, compared to non-Hispanic populations, showed double the increase in excess AAMR. This can be attributed to multiple reasons, including limited access to resources, social determinants of health, racism, and discrimination [31]. Addressing these disparities will require multi-level strategies, including expanding healthcare access through public-private partnerships, prioritizing lifestyle-based interventions in underserved communities, and integrating screening for obesity and hypertension into culturally competent primary care frameworks. Furthermore, although rural areas showed higher mortality over the study period, we observed higher percentages of excess AAMR in urban areas than in rural areas in 2020.

Prior studies examining mortality attributable to obesity and hypertension in isolation have reported substantially lower age-adjusted mortality rates than those observed in our cohort. Obesity-related AAMRs in the United States rose from 1.8 to 3.1 per 100000 between 2010 and 2020 [32], while hypertension-related cardiovascular mortality, though accelerating after 2012, remained below the combined rates reported here [33]. This contrast suggests that the co-occurrence of obesity and hypertension confers a mortality burden that exceeds either condition in isolation, underscoring the importance of studying these comorbidities together rather than independently.

Limitations

This study has several limitations inherent to analyses of national death certificate data. First, CDC WONDER relies on the accuracy of death certificate reporting, which may be subject to the misclassification or underreporting of obesity, given that it is often underdiagnosed or omitted in clinical documentation, potentially underestimating the true mortality burden. Second, the dataset's retrospective nature limits our ability to establish causality or to account for temporal relationships among obesity, hypertension, and mortality. Third, we were unable to adjust for individual-level variables such as socioeconomic status, healthcare access, medication use, lifestyle factors, or disease severity; notably, unmeasured confounders, including smoking status and diabetes, may influence the observed associations and should be considered when interpreting demographic disparities. The ICD-10 code E66 does not capture BMI or obesity class, precluding stratification by severity and limiting dose-response assessment. As population-level prevalence data were not incorporated, it is not possible to distinguish whether rising mortality reflects increasing disease prevalence or worsening outcomes among affected individuals. The inclusion of both underlying and contributing causes of death may introduce variability in case definition and could overestimate deaths directly attributable to the combination of both conditions. Finally, changes in coding practices over time, including the ICD-10 transition, may have influenced observed trends. Despite these limitations, the use of a large, nationally representative database provides valuable insight into long-term mortality patterns.

## Conclusions

In our retrospective analysis of death certificate data from 1999 to 2020, we found that age-adjusted mortality rates among individuals with both obesity and hypertensive diseases consistently displayed an increasing trend across all demographic groups. The overall rising AAMRs, compounded by the disproportionately high average annual percent changes among White individuals and those aged 15-34, raise serious concerns for the healthcare system. These findings have significant implications for public health policy. Focused interventions are essential to curb the upward trajectory of mortality in this population, as early intervention can greatly help tackle the dual burden of these two largely preventable conditions.
